# Identification of the *Staphylococcus aureus* endothelial cell surface interactome by proximity labeling

**DOI:** 10.1128/mbio.03654-24

**Published:** 2025-03-31

**Authors:** Marcel Rühling, Fabio Schmelz, Alicia Kempf, Kerstin Paprotka, Martin J. Fraunholz

**Affiliations:** 1 Chair of Microbiology, Julius-Maximilians-University Würzburg, Würzburg, Germany; MedImmune, Gaithersburg, Maryland, USA

**Keywords:** *Staphylococcus aureus*, host receptor identification, proximity biotinylation

## Abstract

Virulence strategies of pathogens depend on interaction with host cells. The binding and activation of receptors located on the plasma membrane are crucial for the attachment to or pathogen internalization by host cells. Identifying host cell receptors is often difficult, and hence, the identity of many proteins that play important roles during host-pathogen interaction remains elusive. We developed a novel proximity labeling approach by decorating the opportunistic pathogen *Staphylococcus aureus* with ascorbate peroxidase 2. Upon addition of hydrogen peroxide, the peroxidase initiates proximity biotinylation of *S. aureus* host interaction partners, thereby enabling the identification of these proteins. Here, we demonstrate an endothelial cell surface interactome of 305 proteins, including novel *S. aureus* co-receptors such as neuronal adhesion molecule, protein tyrosine kinase PTK7, melanotransferrin, protein-tyrosine kinase MET, and CD109. Filtering the interactome for validated surface proteins resulted in a list of 89 protein candidates, 53% of which were described to interact with *S. aureus* or other pathogens.

**IMPORTANCE:**

*Staphylococcus aureus* is an opportunistic pathogen that enters host cells such as epithelial or endothelial cells. Intracellular pathogens have been observed *in vivo* and are thought to serve immune evasion, avoidance of antibiotic treatment, and chronicity of infection. Thus, it is important to understand the mechanisms by which the bacteria are internalized by host cells; however, screening for pathogen-host receptors is difficult. Here, we developed a novel proximity labeling approach, which enabled the identification of several previously unknown host receptors of *S. aureus* that are engaged during a rapid uptake pathway for the bacteria.

## INTRODUCTION

Pathogens interact with host cell surfaces through a variety of mechanisms that facilitate their entry, survival, and replication within the host. These interactions are crucial for the establishment of infections and can vary significantly among different types of pathogens, including bacteria (1), viruses (2), and protozoa (3). Thereby, pathogens first need to adhere to host cells, which are often mediated through specific interactions with cell surface receptors. For example, many bacteria possess adhesins—surface proteins that bind to host cell receptors, allowing them to colonize, e.g., epithelial surfaces (1). Adherence is a prerequisite for pathogen entry into host cells. Bacteria mainly are internalized either actively inducing their uptake—such as through injection of effectors via specialized secretion systems, termed trigger-type phagocytosis, or through the zipper-type mechanism, which is mediated by receptor-driven actin polymerization (4). Intracellular localization has beneficial effects for the bacteria such as immune evasion or enhanced resistance to antibiotics (5–8). Intensive research has enabled the identification of many receptors over the last decades (1, 9–12). Nevertheless, reliable and efficient methods for identifying host-pathogen interaction partners in their entirety are lacking.

*Staphylococcus aureus* is an opportunistic facultative intracellular pathogen that causes diseases ranging from soft tissue and skin infections to sepsis and toxic shock (13–15). We know today that *S. aureus* possesses intracellular virulence and survival strategies in either phagocytes or even non-phagocytic cells that readily internalize *S. aureus* (16, 17). Invasion of the latter, for example, epithelial or endothelial cells, is mediated by α_5_β_1_ integrins on host cells that bind via fibronectin to fibronectin-binding proteins, which are covalently anchored in the staphylococcal cell wall (10). To date, several entry receptors for staphylococcal invasion have been described, indicating a complex interface between pathogen and host cells (18–25).

We recently described that *S. aureus* can be internalized by host cells via a rapid pathway that takes place within minutes after the bacteria contact the host cell plasma membrane. This unique pathway depends on host cell Ca^2+^ signaling as well as the lysosomal enzyme acid sphingomyelinase (ASM). We hypothesized that the rapid internalization of *S. aureus* by host cells required interaction with a specific subset of host proteins (26).

The identification of host receptors for pathogens is challenging. Genetic approaches, which are frequently based on RNAi or CRISPR technologies, enable high throughput screening for infection-relevant proteins (27). However, these methods are limited to proteins that measurably reduce the cellular uptake of the pathogen. Moreover, a genetic knock-out may permanently alter the composition of cell surface signaling complexes and may not necessarily constitute direct interaction partners of the receptor complex in question. Cross-linking approaches more reliably identify direct host-pathogen interaction partners. Thereby, artificial cross linkers are used to couple proteins on the pathogen surface with host cell receptors (28). However, interaction partners that do not directly bind to the pathogen but may serve important functions during infection, likely are missed.

Here, we present a novel, alternative technique for the identification of host cell proteins in direct proximity to the adhered pathogen, which is based on proximity biotinylation using ascorbate peroxidase 2 (APEX2). APEX2 is an enzyme that enables proximity biotinylation with high temporal resolution (29), enabling the identification of interaction partners with shorter labeling pulses compared to other techniques such as BioID or TurboID (30, 31).

Using this technique, here, we identified and validated neuronal adhesion molecule (NRCAM), protein tyrosine kinase PTK7, melanotransferrin (MFI2), the protein-tyrosine kinase MET, and CD109 as novel *S. aureus* co-receptors.

## RESULTS

### Decorating *S. aureus* with APEX2

**Fig 1:**
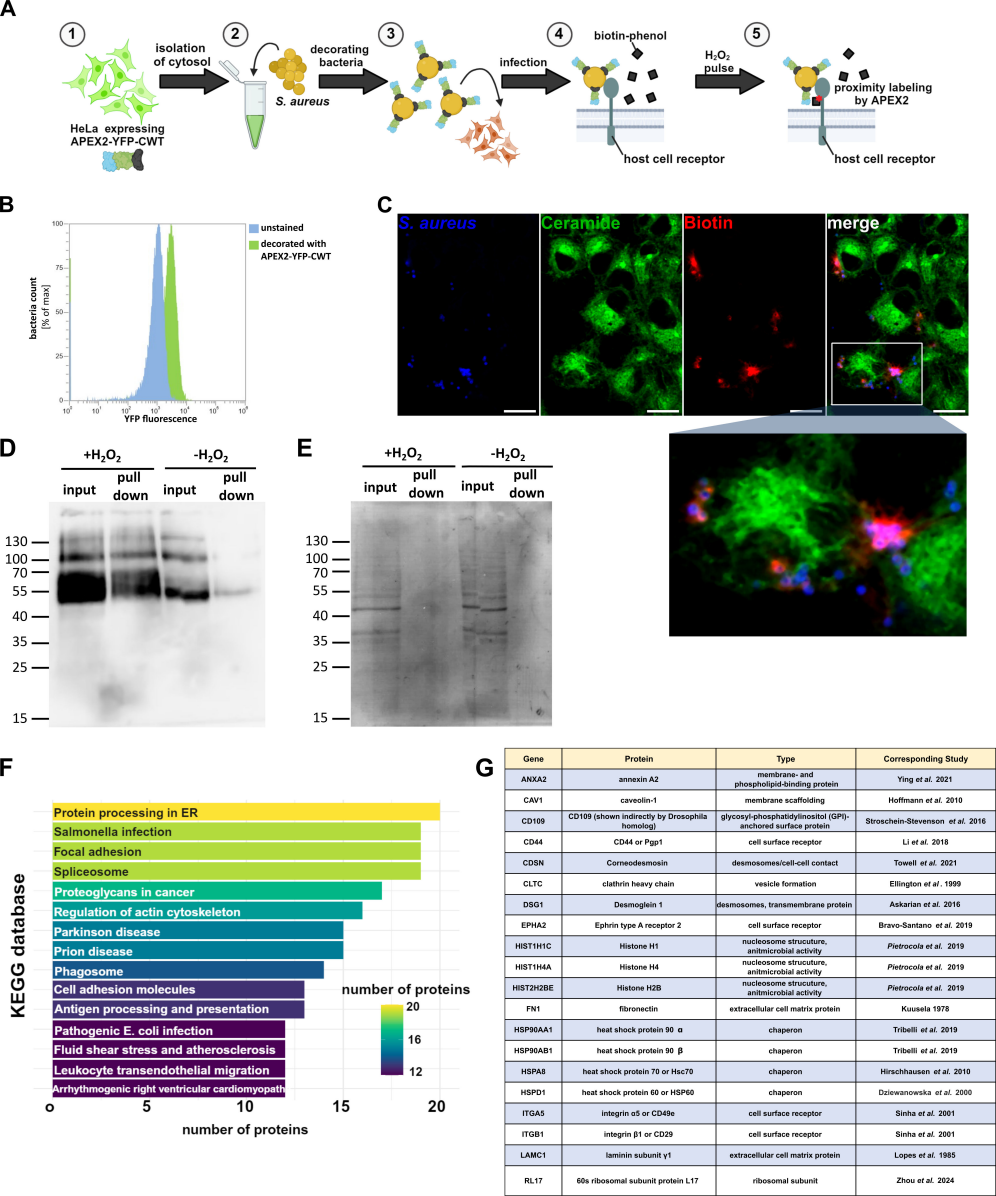
APEX2-based proximity labeling enables the detection of *S. aureus*-host interactome. (A) The cytosol of HeLa expressing the reporter APEX2-YFP-CWT was isolated (1) and used to decorate *S. aureus* JE2 *in vitro* (2). Human lung microvascular endothelial cells (HuLECs) were infected with the bacteria for 15 min (3), and proximity labeling of host receptors was initiated by the addition of biotin phenol (4) and a 1 min pulse of H_2_O_2_ (5). (B) APEX2-YFP-CWT is attached to the *S. aureus* surface. *S. aureus* was treated with a cytosol preparation of the APEX2-YFP-CWT cell line or left untreated. Bacteria were analyzed for YFP fluorescence via flow cytometry. (C) APEX2 decoration of *S. aureus* enables local biotinylation. HeLa was infected with APEX2-decorated bacteria in the presence of biotin phenol for 15 min. Biotinylation was induced by a 1 min H_2_O_2_ pulse, and samples were fixed and stained for biotin via streptavidin-PE, α-NH_2_-ω-N_3_-C_6_-ceramide via BODIPY-FL-DBCO, and *S. aureus* via an anti-*S*. *aureus* antibody. Scale bars: 15 µm. (D and E) Host proteins are biotinylated by APEX2-decorated bacteria and can be isolated by streptavidin pull-down. HuLEC was infected with APEX2-decorated bacteria in the presence of biotin phenol. Biotinylation was induced by a 1 min H_2_O_2_ pulse, or samples were left untreated. Cells were lysed, and biotinylated proteins were isolated by streptavidin pull-down. Lysates and pull-down fractions were analyzed by anti-biotin western blot (D) or Ponceau S staining (E). (F) *S. aureus*-host interaction partners are associated with infection-related pathways. Proteins that were identified by APEX2-based proximity labeling (*n* = 3) were analyzed by pathfindR and categorized with the Kyoto Encyclopedia of Genes and Genomes database. The number of genes associated with the most abundant categories is shown. (G) APEX2 proximity labeling identifies known *S. aureus* interacting proteins. The *S. aureus* host interactome identified by proximity labeling was searched for proteins that are already known for interacting with *S. aureus* (10, 18, 19, 21–25, 34–40).

To identify novel host cell receptors interacting with *S. aureus*, we designed an APEX2-based proximity labeling approach (Fig. 1A), whereby we decorated *S. aureus* with a fusion protein consisting of APEX2, the fluorescent marker yellow-fluorescent protein (YFP), and the cell wall-targeting (CWT) domain of the staphylolytic protease lysostaphin, which binds to the staphylococcal cell wall with high affinity (32). The construct was cytosolically produced in HeLa cells, isolated, and subsequently used to decorate the bacteria. Efficient attachment of APEX2 to the staphylococcal surface was validated by flow cytometry, where we observed a higher YFP mean fluorescence of bacteria treated with cytosol containing APEX2-YFP-CWT (Fig. 1B). Next, we performed infection of unmodified HeLa cells with decorated bacteria in the presence of biotin phenol. Proximity biotinylation was initiated by the addition of H_2_O_2_, the reaction was stopped with a quencher solution, and samples were fixed. We stained biotin with streptavidin-phycoerythrin (PE) and *S. aureus* with an anti-*S*. *aureus* antibody, while we used a fixable ceramide analog (33) to visualize host cells (Fig. 1C). As expected, biotin was detected in close vicinity to the bacteria, indicating that bacteria-bound APEX2 is functional and enables proximity biotinylation of host cell surface proteins.

In order to identify potential *S. aureus* receptors on the surface of human lung microvascular endothelial cells (HuLECs), we infected the cells with APEX2-decorated *S. aureus*, initiated proximity biotinylation with H_2_O_2_, and isolated biotinylated proteins by streptavidin pull-down. We detected significantly more biotinylated proteins in pull-down fractions when H_2_O_2_ was added during infection when compared to the untreated control (Fig. 1D and E). Biotinylated proteins were also detected in cell lysates (input) not treated with H_2_O_2_, even though to a lower extent than in the presence of H_2_O_2_. This can be explained by residual biotin phenol, which is removed by size exclusion before the streptavidin pull-down.

### Proximity labeling identifies NRCAM, PTK7, MFI2, CD109, and MET as ASM-dependent coreceptors of *S. aureus*

In total, mass spectrometry (MS) of the pull-down fractions identified 305 potential *S. aureus* interaction partners in three biological replicates (File S1). Pathway analysis identified “*Salmonella* infection” as well as “focal adhesions” as most enriched terms (Fig. 1F, see Fig. S1A for statistics), indicating that infection-relevant proteins were detected with our approach since focal adhesions are well-known cellular entry sites for *S. aureus* (10). StringDB detected a highly connected protein network (protein-protein interaction [PPI] enrichment *P*-value < 1 × 10^−16^), along with the accumulation of several infection-related proteins (Fig. S1B).

Of the 305 identified proteins, at least 20 were previously reported to interact with *S. aureus*, thereby corroborating our methodology (Fig. 1G).

Recently, we described that *S. aureus* is internalized by host cells via a rapid pathway that depends on host cell Ca^2+^ signaling and the lysosomal enzyme ASM (26). This rapid pathway predominantly mediates host cell entry early during infection. We hypothesized that rapid internalization of *S. aureus* by host cells required interaction with a specific subset of host molecules, which we set out to identify with the proximity labeling approach.

**Fig 2:**
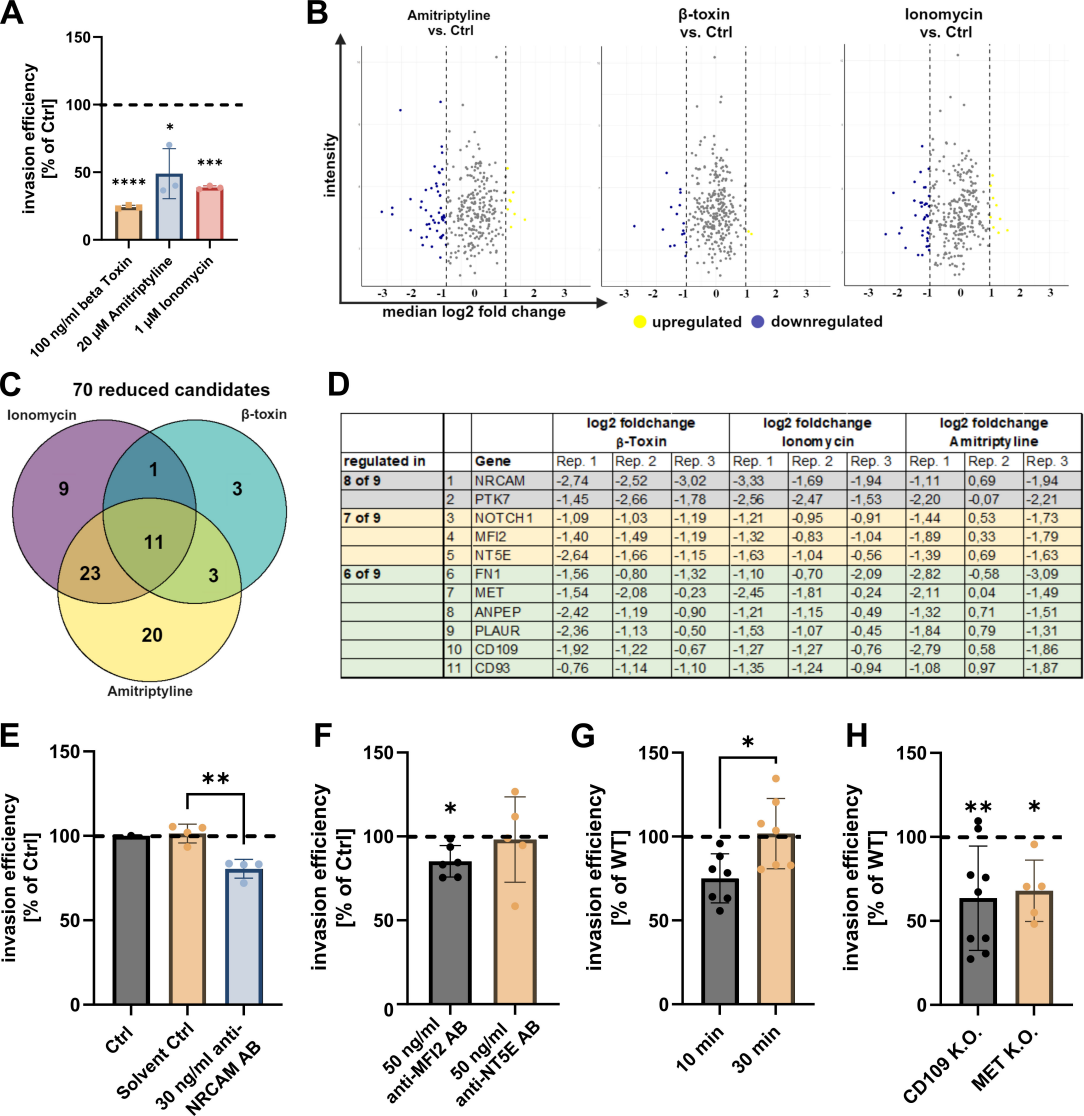
Identification of ASM-dependent host co-receptors for *S. aureus* by an APEX2-based proximity labeling screen. (A) Decorated bacteria are still internalized by host cells in an ASM-dependent manner. HuLEC was treated with amitriptyline, ionomycin, and β-toxin and then infected with APEX2-decorated *S. aureus* JE2. Invasion efficiency was determined by lysostaphin protection assay and colony-forming units (CFU) recovery assays. Numbers of bacteria internalized by treated cells were normalized to untreated controls (set to 100%, dotted line). (B–D) Establishment of the ASM and Ca^2+^-dependent host-pathogen interactome. HuLEC was pretreated with either amitriptyline, the bacterial SMase β-toxin, and ionomycin or left untreated. Cells were infected with APEX2-decorated *S. aureus*, and interaction partners were determined by proximity labeling. (B) Abundance of individual proteins was compared to untreated controls, and median log2 fold changes were determined (*n* = 3). (C) Proteins with a median log2 fold change ≤−1 were selected as candidate targets for each condition. (D) A list of candidate receptors that were downregulated in at least six of nine replicates (regardless of the treatment) were considered as “consistently reduced.” (E and F) Blockade of NRCAM and MFI2 with antibodies reduces the invasion efficiency of *S. aureus*. HuLEC was pretreated with antibodies targeting NRCAM (E) or melanotransferrin (MFI2), NT5E/CD73 (F), or the respective solvent control. Then, cells were infected with *S. aureus* for 10 min, and invasion efficiency was determined by a lysostaphin protection assay and CFU counting (*n* ≥ 4). (G) *S. aureus* invasion is reduced in a PTK7 knockout (K.O.) cell line. The invasion efficiency of *S. aureus* JE2 after 10 min or 30 min was determined in a HeLa cell line lacking PTK7 and was compared to WT cells (*n* = 7). (H) *S. aureus* invasion is reduced in CD109 and MET K.O. cell lines. The invasion efficiency of *S. aureus* JE2 after 10 min was determined in a HeLa cell line lacking CD109 (*n* = 9), and MET (*n* = 5) and compared to WT cells. Statistics: unpaired Student‘s *t*-test (E and G) or one sample *t*-test (F and H). *P ≤ 0.05, **P ≤ 0.01, ***P < 0.001, and ****P < 0.0001.

Therefore, we blocked the ASM-dependent uptake using the ASM inhibitor amitriptyline, the Ca^2+^ ionophore ionomycin, or by the removal of the ASM substrate sphingomyelin from the plasma membrane by treatment with the bacterial sphingomyelinase β-toxin. We next infected the cells with APEX2-decorated bacteria. Despite their APEX2 decoration, the bacteria were still efficiently internalized by host cells (Fig. S2A), and invasion was sensitive to inhibitor treatments as observed for undecorated bacteria (Fig. 2A). Furthermore, the removal of sphingomyelin had no effect on bacterial adherence to host cells (Fig. S2B), excluding that changes in biotinylation intensity arise from lower numbers of bacteria that adhered to host cells in treated samples.

Mass spectrometric comparison of treated samples to the untreated control (Fig. 2B; File S2) revealed 11 proteins that were found to decrease in their abundance in all treatment conditions (Fig. 2C and D).

These included NRCAM, protein tyrosine kinase 7 (PTK7), 5′-nucleotidase NT5E (also called CD73), melanotransferrin (MFI2), neurogenic locus notch homolog protein 1 (NOTCH1), the protein-tyrosine kinase MET, and CD109.

To validate our findings, we treated HuLEC with an anti-NRCAM antibody prior to infection. This reduced rapid *S. aureus* invasion by ~20% when compared to the control (Fig. 2E). While antibody blocking of MFI2 also slightly affected invasion, an antibody directed against NT5E failed to do so (Fig. 2F). A HeLa PTK7 K.O. cell pool (Fig. S2C) showed decreased *S. aureus* invasion for short (10 min) but not for longer (30 min) infection times, suggesting involvement of PTK7 in the rapid Ca^2+^-/ASM-dependent internalization of *S. aureus* (Fig. 2G). Similarly, we observed a decreased internalization of *S. aureus* in cells lacking CD109 or the tyrosine-protein kinase MET at 10 min p.i. (Fig. 2H; Fig. S2D). In summary, here, we validated NRCAM, PTK7, MFI2, CD109, and MET as *S. aureus* host cell entry factors. The remaining candidates will have to be evaluated in future studies.

### Filtering for validated surface proteins increases the hit rate for *S. aureus* receptors

**Fig 3:**
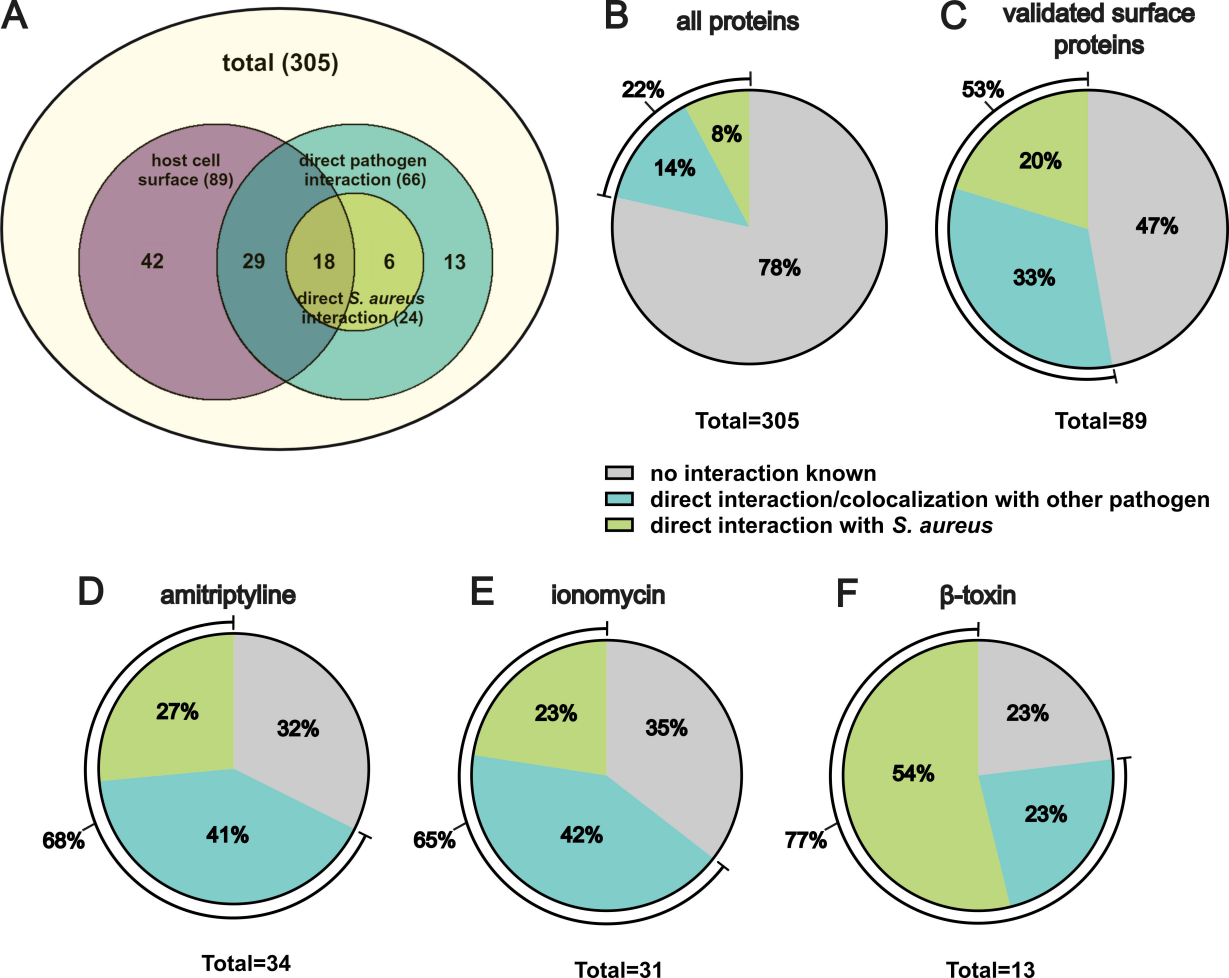
Enrichment of proteins with known pathogen interaction by selecting validated surface proteins. Among the 305 *S*. *aureus* interaction partner candidates identified in the APEX2 screen, 89 were selected as validated surface proteins (41). The data set was then analyzed for proteins that were known to directly interact with or localize to pathogens (42) or *S. aureus* (24) (A) including receptors validated in this study. The proportion of proteins known to interact/colocalize with pathogens or *S. aureus* among all candidates (B) or candidates validated as surface protein (C) was determined. Validated surface proteins that were decreased upon amitriptyline (D), ionomycin (E), or β-toxin (F) treated samples during proximity labeling were analyzed for colocalization/interactions with pathogens or *S. aureus* (including proteins validated in the present study).

We next selected proteins among the 305 candidates that are validated surface proteins according to a cell surface protein atlas (41). We identified 89 proteins that were detected by proximity labeling and possess validated host surface localization from which many are known interaction partners of pathogens including *S. aureus* (Fig. 3A; see File S1 for corresponding references). This analysis includes receptors validated in the present study by antibody blocking or genetic ablation. Selecting for host cell surface proteins increased the hit rate for known interaction partners of pathogens/*S. aureus* (Fig. 3B and C; File S1). The unfiltered data set contained 22% of known pathogen interaction partners (8% for *S. aureus*, 14% for other pathogens; Fig. 3B). This proportion increased to 53% after selecting validated surface proteins (20% for *S. aureus* and 33% for other pathogens; Fig. 3C).

This proportion was even higher for candidate receptor proteins that were decreased upon blocking the rapid internalization pathway by amitriptyline (Fig. 3D), ionomycin (Fig. 3E), or β-toxin (Fig. 3F). In all three conditions, proximity labeling detected predominantly proteins with already known pathogen interaction.

## DISCUSSION

The identification of receptors by genetic approaches, such as RNAi or CRISPR (27), is restricted to receptors that reduce the cellular uptake of the pathogen of interest, while other proteins that are not directly involved in internalization, but might have other functions during infection, are missed. Moreover, the reduced invasion of a pathogen after ablation of a host gene does not necessarily identify the respective gene as a host cell receptor, as it may serve other cellular functions important for pathogen uptake such as regulation of membrane composition or actin organization. Cross-linking approaches also enable the identification of receptors (28), but they require molecular interaction between host receptors and the pathogen surface. Thus, receptors that are not directly bound to the pathogen surface but might be recruited to the pathogen entry site as a co-receptor are not detected. Additionally, the analysis of cross-linked proteins from complex samples, such as whole-cell lysates, is challenging (43).

Here, we present a novel technique for the identification of receptors, which is based on proximity biotinylation. Compared to other approaches that previously were used to identify host cell receptors, the APEX2-based method has important advantages. The identification of proteins by proximity labeling requires close vicinity to the pathogen but does not need an interaction at the molecular level. Moreover, proximity biotinylation is not restricted to receptors that are directly involved in the internalization of the pathogen. Hence, we think that the APEX2-based approach enables the detection of host proteins that are present at host-pathogen contact sites in their entirety. However, APEX2-based biotinylation also detects bystander proteins, which are randomly localized at the site of infection.

We hypothesized that a specific set of host cell surface receptors is required for the rapid uptake of *S. aureus* by endothelial cells (26). Since the identification of receptors that are involved in fast invasion processes is exceptionally demanding, we designed an assay for proximity labeling of host surface receptors using APEX2-decorated bacteria (Fig. 1A). Compared to other techniques such as BioID and TurboID (30, 31), APEX2 allows for proximity biotinylation with high temporal resolution (29). Whereas BioID and TurboID are biotin ligases, the mechanism of action differs from that of peroxidase such as APEX2. Biotin ligases use biotin as a substrate to produce biotinoyl-5′-AMP that reacts with lysine residues, while peroxidases generate reactive radicals that tag tyrosine residues from biotin phenol (44, 45). Using the naturally occurring biotin as a substrate, biotin ligases might be more suitable for *in vivo* applications (45). Our approach identified 305 candidate proteins (File S1), of which many were shown to interact with *S. aureus* such as α_5_β_1_ integrins (10), fibronectin (34), CD44 (18), annexin 2 (19), Hsp60 (20), Hsc70 (21), Hsp90 (22), desmoglein 1 (23), corneodesmosin (24), laminin (35), ephrin type A receptor 2 (25), and others. However, we did not detect all host receptors known to interact with *S. aureus*; for instance, tumor necrosis factor α receptor 1, which binds to staphylococcal protein A, was not found in streptavidin pull-down (46). This remains unclear but may be caused by the low abundance of TNFR1 on the surface of HuLEC, the absence of accessible tyrosine residues for biotinylation, or steric hindrance induced by APEX2 decoration.

Of the 305 protein candidates, we stringently selected proteins (Fig. 2D) that were found reduced in conditions that block the rapid ASM-dependent internalization pathway. Of the resulting 11 proteins, we validated PTK7, NRCAM, MFI2, CD109, and MET as novel (co)receptors for ASM-dependent host cell entry of *S. aureus* (Fig. 2E through H). PTK7, an inactive tyrosine kinase, was previously linked to extracellular matrix rearrangement, cytoskeleton, and focal adhesions (47, 48) and was required exclusively for the early internalization of *S. aureus* (Fig. 2G).

NRCAM possesses fibronectin-like as well as immunoglobulin-like domains (49) that could interact with staphylococcal fibronectin-binding proteins or protein A, respectively. The tyrosine kinase MET, which we found involved in *S. aureus* invasion, is a well-known host cell receptor of *Listeria monocytogenes* (9). Moreover, the *Drosophila melanogaster* homolog of CD109, TepIII, was previously shown to play a role in the phagocytosis of *S. aureus* (36). We also detected reduced interaction of *S. aureus* with other proteins upon blocking ASM-dependent internalization such as NOTCH1, alanyl aminopeptidase (ANPEP), urokinase receptor (PLAUR), and CD93, which remain to be validated by further experimentation. CD93 was associated with phagocytosis (50) as well as cell adhesion (51) and possesses a C-type lectin domain (52), a motif known to interact with pathogen-associated carbohydrate structures (53). Macrophages and neutrophils lacking PLAUR showed a decreased phagocytosis of the bacterium *Burkholderia pseudomallei*, although a direct interaction was not demonstrated (54). Furthermore, PLAUR was shown to affect the affinity of α_5_β_1_ integrins, an important *S. aureus* host cell entry receptor (10), for fibronectin (55), which could directly influence the binding of staphylococcal fibronectin-binding proteins to integrin receptors. Likewise, NOTCH1 was shown to interact with β_1_ integrins (56, 57), suggesting a possible influence of NOTCH1 on integrin-mediated uptake of *S. aureus*. Here, we suggest that next to primary receptors such as α_5_β_1_ integrins or CD44, which initially interact with the pathogen (10, 18), PTK7, NRCAM, MFI2, CD109, and MET, are accessory receptors, whose interaction with the bacteria modifies and accelerates the internalization. Hence, the blockade of each receptor had a moderate yet significant impact on *S. aureus* internalization (Fig. 2E through H). *S. aureus-*mediated release of ASM by lysosomal exocytosis may facilitate the recruitment of NRCAM, PTK7, and MFI2 or other receptors, e.g., via the formation of ceramide-enriched platforms. Supporting this model, blocking ASM-dependent internalization did not reduce the interaction of *S. aureus* with known primary receptors such as α_5_β_1_ integrin or CD44.

ASM dependence was demonstrated for the host cell entry of a variety of pathogens. Hence, we suggest that the surface molecules identified by APEX2-based proximity labeling represent interesting candidate receptors for other bacteria and viruses. For instance, MET and ANPEP, which also were identified on the screen, are known entry factors for *Listeria* (9) and human coronavirus (58), respectively. Interestingly, a rapid ASM-dependent uptake previously was demonstrated for adenovirus (59), suggesting that multiple pathogens use the presented strategy.

In our study, the CWT domain enabled the attachment of APEX2 to *S. aureus*. Since the interaction between CWT and the staphylococcal cell wall is highly specific, the decoration of other pathogens with APEX2 would require alternative proteins, such as APEX2-conjugated antibodies or bacteriophage receptor-binding proteins (60). Furthermore, it is unclear to which extent the presence of the APEX2 construct on the staphylococcal surface affects interaction with host proteins. However, APEX2-decorated bacteria were still cell invasive (Fig. S2A), suggesting that interaction with host cell entry receptors is not or only negligibly affected. Proximity labeling is prone to detection of false-positive candidates either due to biotinylation of highly abundant by-stander proteins or unspecific binding during streptavidin pull-down. For instance, several ribosomal subunits and histones were detected by proximity labeling in our data. Although some studies suggest an interaction between *S. aureus* and histones (40) or the ribosomal subunit RL17 (37), we think that these interactions are rather unlikely to happen on the surface of endothelial host cells.

The CWT domain and YFP, important constituents of the APEX2 decoration construct, create a distance between the staphylococcal surface and APEX2, which we estimate to a maximum of 10 nm by generating a model of the construct (Fig. S3D). However, we do not think that this will affect the results of proximity biotinylation since (i) the area of pathogen-host interactions should be clearly larger, and (ii) the labeling radius of APEX2 was estimated to ~10–20 nm (61).

Nevertheless, we reached a hit rate of ~20% known *S. aureus* receptors after filtering for surface resident proteins (Fig. 3C). Hence, proteins detected by our proximity labeling approach have identified additional potential *S. aureus* surface interaction partners, which is especially supported by the high proportion of proteins known to interact with other pathogens. Our data set thus indicates that diverse pathogens use a similar subset of molecules to interact with host cells. It will be interesting to compare host interactomes of different pathogens in future studies.

## MATERIALS AND METHODS

### Cell culture

HeLa (ATCC CCL-2) was cultivated in Roswell Park Memorial Institute (RPMI) 1640 medium with GlutaMAX (Gibco, Cat. no. 72400054) supplemented with 10% (vol/vol) heat-inactivated (56°C at 30 min) fetal bovine serum (FBS; Sigma Aldrich, Cat. no. F7524) and 1 mM sodium pyruvate (Gibco, Cat. no. 11360088).

HuLEC-5a (ATCC CRL-3244) was cultured in MCDB131 medium (Gibco, Cat. no. 10372019) complemented with microvascular growth supplement (Gibco, Cat. no. S00525), 2 mM GlutaMAX (Gibco, 35050061), 5% (vol/vol) heat-inactivated (56°C at 30 min) FBS, 2.76 µM hydrocortisone (Sigma Aldrich, Cat. no. H0888), 0.01 ng/mL human epidermal growth factor (Pep Rotech, AF-100–15), and 1× penicillin-streptomycin (Gibco, Cat. no. 15140122).

HuLEC was detached by StemPro Accutase (Gibco, Cat. no. A1110501) and seeded at the indicated density 2 days prior to the experiment, whereas HeLa was detached with TrypLE (Gibco, Cat. no. 12604013) and seeded 1 day prior to the experiment.

### Generation of HeLa K.O. cell lines

The generation of K.O. cell lines is based on a previous protocol (62). Single guide RNAs (sgRNAs; Table 1) were designed with the CHOPCHOP online tool (chopchop.cdu.uib.no) and synthesized as primer pairs (Sigma Aldrich). After phosphorylation with T4 nucleotide kinase (ThermoFisher, Cat. no. ER0031), the sgRNAs were cloned into the BbsI sites of pSpCas9(BB)-p2A-GFP (AddGene #48138 [62]) via forced ligation by BbsI (ThermoFisher, Cat. no. ER1012) and T4 Nucleotide ligase (ThermoFisher, Cat. no. EL0011). The constructs were then transformed in *Escherichia coli* DH5α (ThermoFisher, Cat. no. EC0112) and subsequently sequence verified.

**TABLE 1:** Oligonucleotides used in this study

Purpose	Name	Sequence (5′→ 3′)
Gene deletion	PTK7-sgRNA 2 rv	AAACTGCATTGCGTGGCCGGACCT
	PTK7-sgRNA 2 fw	CACCAGGTCCGGCCACGCAATGCA
	PTK7-sgRNA1 rv	AAACACCGACAACCCAATGGTCTG
	PTK7-sgRNA1 fw	CACCCAGACCATTGGGTTGTCGGT
	CD109-sgRNA 1 rv	AAACATATCCAAGTGACCGTGACG
	CD109-sgRNA 1 fw	CACCCGTCACGGTCACTTGGATAT
	CD109-sgRNA 2 rv	AAACGCGGAAATATTAACTGCCGT
	CD109-sgRNA 2 fw	CACCACGGCAGTTAATATTTCCGC
	MET-sgRNA 1 rv	AAACTTCGCCGAAATACGGTCCTA
	MET-sgRNA 1 fw	CACCTAGGACCGTATTTCGGCGAA
	MET-sgRNA 2 rv	AAACACAAGTATTTCGCCGAAATA
	MET-sgRNA 2 fw	CACCTATTTCGGCGAAATACTTGT
Cloning	infus-CWT-r	GAGGTTGATTATCATATGACTAGTTCACTTTATAGTTCCCCAAAG
	cYFP-f	ATGGTGAGCAAGGGCGAGGAGCTG
	his1-Flag-f	CATCATCATCATCACGGAGCGGACTACAAGGATGACGACGATAAGG
	his2-Inf-f	GACTAGCCTCGAGGTTTAAACCACCATGGCACATCATCATCATCATCACGGAGCG
	Apex-YFP-infus-r	CGCCCTTGCTCACCATCTCGAGCCGTCCAGGGTCAGGCGCTCC

HeLa cells were transfected with 1 µg each of two distinct sgRNA constructs using JetPrime (Polyplus) following the manufacturer’s instructions. After 36–48 h, cells were sorted for strong green-fluorescent protein (GFP) expression in a fluorescence-activated cell sorting (FACS) Aria III (BD Bioscience). The resulting cell pools were cultivated and tested for loss of expression of the respective sgRNA target by western blotting.

The following antibodies were used: mouse anti-PTK7 monoclonal IgG1 (bio-techne, Cat. no. MAB4499) with a horse radish peroxidase (HRP)-conjugated anti-mouse antibody (SantaCruz, Cat. no. sc-516102) and rabbit anti-cMet monoclonal IgG (Thermo Fisher, Cat. no. 700261) with an HRP-conjugated anti-rabbit antibody (Biozol, Cat. no. 111-035-144).

### Generation of APEX2 construct *pLV-APEX2-YFP-CWT*

YFP-CWT was amplified from pLVTHM-YFP-CWT (63) with oligonucleotides cYFP-f and infus-CWT-r, thereby introducing overhangs suitable for InFusion cloning

Next, we amplified FLAG-APEX from pLX304-Flag-APEX2-NES (64) with his1-Flag-f and APEX-YFP-infus-r, a subsequent reamplification with his2-Inf-f and APEX-YFP-infus-r thereby introducing a N-terminal 6× His-tag. Subsequently, we performed an InFusion reaction with PmeI/SpeI-opened pLVTHM vector (65) as well as 6xHis-FLAG-APEX2 (886 bp) and YFP-CWT (1,089 bp). The resulting vector was termed pLV-6xHis-FLAG-APEX2-YFP-CWT.

Constructs were transformed into *E. coli* DH5α, and sequences were validated. Plasmid preparations were performed using standard laboratory procedures. VSV-G-pseudotyped lentiviral particles were generated by transfecting with each lentiviral vector as well as the plasmids pMD2.G (VSV G) and psPAX2 following the protocol of (65), and target cells were transduced as described previously (63).

### *S. aureus* culture

*S. aureus* liquid cultures were either grown in 37 g/L brain heart infusion (BHI) medium (Thermo Fisher, Cat. no. 237300). *E. coli* liquid cultures were grown in Luria-Bertani (LB) medium (10 g/L tryptone/peptone [Roth, Cat. no. 8952.4], 5 g/L yeast extract [Roth, Cat. no. 2363.2], and 10 g/L NaCl [Sigma Aldrich, Cat. no. S5886]).

For agar plates, 15 g/L agar (Otto Norwald, Cat. no. 257353) was added to either tryptic soy broth (TSB) for *S. aureus* or lysogeny broth (LB) for *E. coli*.

Media and plates were supplemented with appropriate antibiotics (5 µg/mL erythromycin [Sigma Aldrich, Cat. no. E5389], 10 µg/mL chloramphenicol [Roth, Cat. no. 3886.2], and 100 ng/mL carbenicillin [Roth, Cat. no. 6344.2]).

### *S. aureus* infections

Host cells were seeded in 6-well plates (2 × 10^5^ cells per well), 12-well plates (1 × 10^5^ cells per well), or 24-well plates (0.5 × 10^5^ cells per well) either 1 day (HeLa^−^) or 2 days (HuLEC) prior to the experiment. Host cells were washed thrice with Dulbecco’s phosphate-buffered saline (DPBS) and infection medium (MCDB131 medium [Gibco, Cat. no. 10372019] complemented with GlutaMAX [Gibco, 35050061] and 10% [vol/vol] heat-inactivated [56°C at 30 min] FBS) was added. If indicated, cells were pretreated with compounds prior to infection (for details about treatments, see Table 2). For experiments involving blocking of receptors by antibodies, respective solvent controls were implemented (final concentrations in infection medium): 0.002% (wt/vol) NaN_3_ (Sigma Aldrich, Cat. no. S2002), 5% glycerol (Roth, Cat. no 3783.2) in 10% DPBS for 30 ng/mL anti-NRCAM antibody as well as 0.01% (wt/vol) NaN_3_ for 50 ng/mL anti-NT5E, and 50 ng/mL anti-MFI2 antibodies.

**TABLE 2:** Host cell treatment with various compounds

Compound	Supplier/cat. no.	Preincubation time	Removal prior to infection?
30 ng/mL anti-NRCAM antibody	Proteintech/21608–1-AP	75 min	Removed prior to infection
50 ng/mL anti-NT5E antibody	Thermo Fisher/41–0200	75 min	Removed prior to infection
50 ng/mL anti-MFI2 antibody	Biozol/BLD-363101	75 min	Removed prior to infection
Amitriptyline	Sigma Aldrich/A8404	75 min	Present during infection
Ionomycin	Sigma Aldrich/I0634	75 min	Present during infection
β-toxin	Sigma Aldrich/S8633	75 min	Removed prior to infection

For all infections, the *S. aureus* strain JE2 was used (66). An *S. aureus* overnight culture grown in BHI containing the appropriate antibiotics was diluted to an OD_600_ = 0.4 in the same medium. The culture was grown to an OD_600_ = 0.6–1.0, and 1 mL bacterial suspension was harvested by centrifugation and washed twice with DPBS (Gibco, Cat. no. 14190169). The bacteria were resuspended in the infection medium. The number of bacteria per milliliter in the suspension was determined with a Thoma counting chamber, and the multiplicity of infection (MOI) was determined. If not indicated otherwise, an MOI = 10 was used for infections.

The infection was synchronized by centrifugation 800 × g/8 min/RT (end of the centrifugation: *t* = 0). To determine bacterial invasion, the infection was stopped after 30 min (unless indicated otherwise) by removing extracellular bacteria with 20 µg/mL lysostaphin (AMBI, Cat. no. AMBICINL) in the infection medium for 30 min. Then, host cells were washed thrice with DPBS, lysed by addition of 1 mL/well (12-well plate) or 0.5 mL/well (24-well plate) Millipore water, and the number of bacteria in lysates was determined by plating serial dilutions (10^−1^, 10^−2^, and 10^−3^) on TSB agar plates. Plates were incubated overnight at 37°C, and CFU was enumerated. To determine invasion efficiency, the number of bacteria determined in tested samples was normalized to untreated controls (set to 100%).

### Decoration of *S. aureus* with APEX2-YFP-CWT

HeLa cells expressing APEX2-YFP-CWT were seeded into 12 tissue culture dishes (Ø150 mm) with a density of 6 × 10^5^ cells per dish and cultured for 5 days (medium was exchanged after 2–3 days). Isolation of the cytoplasm was based on a previously published protocol (67). Shortly, cells were washed thrice with DPBS, and then, cells were scraped from the substratum in 4 mL cold DPBS per dish. The cell suspension for three dishes was pooled, and samples were centrifuged at 800 × *g* for 7 min at 4°C. The pellet of each cell pool was resuspended in 1 mL buffer B (20 mM HEPES, pH 7.6 [Roth, Cat. no. 6763.3], 220 mM mannitol [Roth, Cat. no. 4175.1], 70 mM sucrose [Roth, Cat. no. 4621.2], 1 mM EDTA [Roth, Cat. no. 8040.2], and 1× protease inhibitor cocktail [Sigma Aldrich, Cat. no. 11873580001]), and cells were incubated for 20 min at 4°C. Subsequently, cells were lysed by a tissue homogenizer, and lysates were cleared by centrifugation 10,000 × *g*/10 min/4°C. Then, lysates were centrifuged in an Optima MAX-XP ultracentrifuge (Beckman Coulter) at 100,000 × g at 4°C for 45 min. The supernatants containing the cytosol of the cell pools were combined and concentrated in an Amicon centrifuge concentrator tube (3 kDa cutoff, Sigma Aldrich, Cat. no. UFC901024) by centrifugation at 4,000 × g/4°C for 30–45 min until a volume of 1.5–2.0 mL was reached.

For decoration of *S. aureus* with APEX2-YFP-CWT, an *S. aureus* overnight culture was grown in BHI medium, freshly diluted to OD_600_ = 0.4 in 10 mL BHI medium, and grown until an OD_600_ = 0.6–1.0 was reached. Three milliliters of the culture was harvested by centrifugation, washed twice with DPBS, and incubated with the concentrated cytoplasm of HeLa APEX2-YFP-CWT for 30 min at RT in an end-over-end rotator. The decorated bacteria were washed once with DPBS and resuspended in 1 mL Hanks’ balanced salt solution (HBSS; Gibco, Cat. no. 14025-100), and the number of bacteria was determined in a Thoma counting chamber. The bacterial suspension was then either used for invasion studies or proximity labeling. To validate the decoration of bacteria, the *S. aureus* suspension was analyzed with an Attune NxT flow cytometer (Thermo Fisher, Attune Cytometric Software v5.2.0) gating for YFP fluorescence (Ex. 488 nm/Em. bandpass 530/30 nm).

### Proximity labeling with APEX2-decorated bacteria

Based on a published protocol (68), we seeded HuLEC in six-well plates with a density of 2.5 × 10^5^ cells per well (three wells per sample) 2 days prior to the experiment. Cells were pretreated with an infection medium containing 1 µM ionomycin, 100 ng/mL β-toxin, or 20 µM amitriptyline for 75 min. Then, we washed the cells thrice with DPBS and added a fresh infection medium containing 1 mM biotin-phenol (Sigma Aldrich, SML2135) as well as amitriptyline and ionomycin. At this stage, we omitted β-toxin. Subsequently, cells were infected with APEX2-decorated *S. aureus* JE2 at an MOI = 50 by centrifuging the bacteria on the host cells at 800 × g for 8 min at RT. After 15 min, cells were washed thrice to remove non-attached bacteria. Then, 1 mL per well HBSS containing 1 mM biotin phenol was applied, and biotinylation was initiated by adding 0.003% (vol/vol) H_2_O_2_ (Sigma Aldrich, Cat. no. H1009) for 1 min. Controls without H_2_O_2_ addition were performed. Immediately, cells were washed once with cold DPBS, and 2 mL per well quencher solution (10 mM sodium ascorbate [Sigma Aldrich, Cat. no. A4034], 5 mM Trolox [Sigma Aldrich, Cat. no. 238813], and 10 mM sodium azide [Sigma Aldrich, Cat. no. S2002]) was applied to stop biotinylation. Then, cells were either fixed with 0.2% glutaraldehyde (Sigma Aldrich, Cat. no. 10333) and 4% paraformaldehyde in PBS (Morphisto, Cat. no. 11762.01000) for 30 min RT and further prepared for microscopy or processed for streptavidin pull-down. Therefore, cells were detached with a cell scraper, and three wells per condition were pooled. Cells were centrifuged at 3,500 × *g* for 7 min at 4°C and were resuspended in 100 µL radioimmunoprecipitation assay (RIPA) buffer (50 mM TRIS-HCl [Sigma Aldrich, Cat. no. T1503], 150 mM NaCl [VWR, Cat. no. 27810.364], 1% [vol/vol] Triton-X 100 [Roth, Cat. no. 3051.4], 0.1 [wt/vol] SDS [Roth, Cat. no. CN30.3], 2.5 [wt/vol] sodium deoxycholate [Roth, Cat. no. 3484.3], and 1× protease inhibitor cocktail [Sigma Aldrich, Cat. no. 11873580001]). After lysis for 30 min at 4°C, samples were centrifuged 14,000 × *g* for 10 min at 4°C. Lysates were loaded to an Amico centrifuge concentrator (3 kDa cutoff, Sigma Aldrich, Cat. no. UFC901024) and centrifuged at 4,000 × *g* for 30 min at 4°C to remove residual biotin phenol until a volume of ~1 mL was reached. The concentrate was diluted with 5 mL DPBS containing 1× protease inhibitor (Sigma Aldrich, Cat. no. 11873580001) and re-concentrated by centrifuging at 4,000 × *g* for 30–45 min at 4°C until a volume of 500–100 µL was reached. The same procedure was repeated with 5 mL RIPA buffer. Next, 40 µL per sample of magnetic streptavidin beads (Thermo Fisher, Cat. no. 88816) was equilibrated with 1 mL per sample RIPA buffer and incubated with the concentrated biotin phenol-free cell lysates at 4°C overnight with permanent rotation.

Next, the beads were washed with (i) 2 × 1 mL RIPA buffer, (ii) 1 × 1 M KCl (Roth, Cat. no. 6781.1), (iii) 1 × 0.1 M Na_2_CO_3_ (Roth, Cat. no. 8563.1), (iv) 2 M urea (Roth, Cat. no. 2317.1) in 10 mM TRIS-HCl, pH 8 (Sigma Aldrich, Cat. no. T1503), and (v) 1× RIPA. Then, beads were resuspended in 50 µL 6× Laemmli (60 mM TRIS-HCl, pH 6.8 [Sigma Aldrich, Cat. no. T1503], 12% [wt/vol] SDS [Roth, Cat. No. CN30.3], 47% [vol/vol] glycerol [Roth, Cat. no. 3783.2], dithiothreitol [DTT; Roth, Cat. no. 4227], and 0.01% [wt/vol] bromophenol blue [Sigma Aldrich, Cat. no. B0126]) containing 2 mM biotin (Thermo Fisher, Cat. no. 29129) and incubated at 98°C for 10 min to eluate biotinylated proteins. The eluates were analyzed via mass spectrometry.

To verify the pull-down of biotinylated proteins, the cell lysates of samples before incubation with streptavidin beads (input) were compared with pull-down fractions by western blot. Therefore, 50 µL input samples was mixed with 10 µL 6× Laemmli, boiled at 98°C/10 min and subsequently, and analyzed by semi-dry western blot. Polyvinylidene difluoride (PVDF) membranes were blocked by 5% milk powder (AppliChem, Cat. no. A0830,5000) in TBS-T, pH 7.5 (50 mM Tris [Sigma Aldrich, Cat No. T1503], 150 mM NaCl [VWR, Cat. no. 27810.364], and 0.05% [vol/vol] Tween [Roth, Cat. no. 9127.2]). Membranes were incubated with an HRP-linked anti-biotin antibody (Cell Signaling, Cat. no. 7076S) overnight and developed with an HR-16-3200 chemiluminescence reader (Intas).

### Mass spectrometry

#### Single-pot, solid-phase-enhanced sample preparation

Samples were processed using an adapted single-pot, solid-phase-enhanced sample preparation protocol (42). Briefly, 200 µL reconstitution solution was added to each sample prepared in 50 µl Laemmli buffer (Life Technologies), resulting in a DTT concentration of 12 mM. Alkylation was performed with 35 mM iodoacetamide. Additional DTT (10 mM) was used for quenching. Equal volumes of two types of Sera-Mag Speed Beads (Cytiva, #45152101010250 and #65152105050250) were combined and washed with water, and 10 µL of the bead mix was added to each sample. A total of 260 µL 100% ethanol was added, and samples were incubated for 5 min at 24°C, 1,000 rpm. Beads were captured on a magnetic rack for 2 min, and the supernatant was removed. Beads were washed twice with 200 µL 80% ethanol (Chromasolv, Sigma) and then once with 1,000 µL 80% ethanol. The on-bead digest was performed with 0.25 µg trypsin (Gold, Mass Spectrometry Grade, Promega) and 0.25 µg Lys-C (Wako) in 100 µL 100 mM ammonium bicarbonate at 37°C overnight. Peptides were desalted using C-18 Stage Tips (69). Each Stage Tip was prepared with three discs of C-18 Empore SPE Discs (3M) in a 200 µL pipette tip. Peptides were eluted with 60% acetonitrile in 0.1% formic acid, dried in a vacuum concentrator (Eppendorf), and stored at −20°C. Peptides were dissolved in 2% acetonitrile/0.1% formic acid prior to nano liquid chromatography tandem mass spectrometry (LC-MS/MS) analysis.

#### Nano LC-MS/MS analysis

Nano LC-MS/MS analyses were performed on an Orbitrap Fusion (Thermo Scientific) equipped with a PicoView Ion Source (New Objective) and coupled to an EASY-nLC 1000 (Thermo Scientific). Peptides were loaded on a trapping column (2 cm × 150 µm ID, PepSep) and separated on a capillary column (30 cm × 150 µm ID, PepSep) both packed with 1.9 µm C18 ReproSil and separated with a 120 min linear gradient from 3% to 30% acetonitrile and 0.1% formic acid and a flow rate of 500 nL/min. Both MS and MS/MS scans were acquired in the Orbitrap analyzer with a resolution of 60,000 for MS scans and 30,000 for MS/MS scans. High-energy collisional dissociation (HCD) fragmentation with 35% normalized collision energy was applied. A top-speed data-dependent MS/MS method with a fixed cycle time of 3 s was used. Dynamic exclusion was applied with a repeat count of 1 and an exclusion duration of 90 s; singly charged precursors were excluded from selection. The minimum signal threshold for precursor selection was set to 50,000. Predictive automatic gain control (AGC) was used with AGC target values of 4 × 10^5^ for MS scans and 5 × 10^4^ for MS/MS scans. EASY-IC was used for internal calibration.

#### MS data analysis

Raw MS data files were analyzed with MaxQuant version 1.6.2.2 (70). Database search was performed with Andromeda, which is integrated in the utilized version of MaxQuant. The search was performed against the UniProt Human Reference Proteome database (June 2022, UP000005640, 79684 entries), an *S. aureus* GenBank database (accession numbers NC_007793, NC_007792, NC_007791, and NC_007790) and a small database containing the APEX2 constructs. Additionally, a database containing common contaminants was used. The search was performed with tryptic cleavage specificity with three allowed miscleavages. Protein identification was under the control of the false discovery rate (FDR; <1% FDR on protein and peptide spectrum match level). In addition to MaxQuant default settings, the search was performed against the following variable modifications: Protein N-terminal acetylation, Gln to pyro-Glu formation (N-term. Gln), and oxidation (Met). Carbamidomethyl (Cys) was set as a fixed modification. Further data analysis was performed using R scripts developed in-house. Label-free quantification (LFQ) intensities were used for protein quantitation (71). Proteins with less than two razor/unique peptides were removed. Missing LFQ intensities were imputed with values close to the baseline. Data imputation was performed with values from a standard normal distribution with a mean of the 5% quantile of the combined log10-transformed LFQ intensities and an SD of 0.1.

For the identification of significantly enriched proteins, median log2 transformed protein ratios were calculated from the three replicate experiments, and boxplot outliers were identified in intensity bins of at least 300 proteins. Log2 transformed protein ratios of sample vs control with values outside a 1.5× (significance 1) or 3× (significance 2) interquartile range, respectively, were considered as significantly enriched in the individual replicates.

### Microscopy

HeLa cells were incubated with 10 µM α-NH_2_-ω-N_3_-C_6_-ceramide (33) for 30 min prior to infection with APEX2-decorated bacteria. After proximity biotinylation, samples were fixed with 0.2% (vol/vol) glutaraldehyde (Sigma Aldrich, Cat. no. 10333) and 4% paraformaldehyde in PBS (Morphisto, Cat. no 11762.01000) for 30 min RT. Cells were permeabilized with 0.2% (vol/vol) Triton X-100 (Thermo Fisher, Cat. no. 28314) for 20 min RT, and α-NH_2_-ω-N_3_-C_6_-ceramide was stained with 2 µM BODIPY-FL-DBCO (Jena Biosciences, Cat. no. CLK-040–05) for 30 min at 37°C. Then, samples were blocked with 10% FBS in PBS for 1 h RT. *S. aureus* was stained with 25 ng/mL anti-*S*. *aureus* antibody (Thermo Fisher, PA1-7246) at 4°C overnight, and an AlexaFluor405-conjugated secondary antibody (Thermo Fisher, Cat. no. A48254) for 1 h at RT. Biotin was stained by 5 ng/mL streptavidin-PE (Becton Dickinson, Cat. no. 554061) at 4°C overnight. Imaging was performed at a confocal SP5 TCS microscopy (Wetzlar, Germany; Software Leica LAS AF Version 2.7.3.9723) with a 40× immersion oil objective (NA1.3) and a resolution of 2,048 × 2,048 pixels.

### R analysis

MS results from APEX2 proximity labeling screens were processed in R studio (72). Candidates that were identified in all three biological replicates were selected for further analysis (in total 354 proteins). First, the targets were categorized by the pathfindR package (73) according to the Kyoto Encyclopedia of Genes and Genomes (74–76). In the input matrix, fold changes and *P*-values were arbitrarily set to 1 and 0.05, respectively. Proteins listed in the category “ribosomes” were removed from the data set for further processing (File S1). Then, the number of candidates in other categories was counted and plotted with ggplot (77).

Log_2_ fold changes (FCs) were calculated based on LFQ detected during MS analysis in treated conditions vs LFQs detected in untreated control infections. The log_2_ FCs were rounded to one decimal place, and a threshold of log_2_FC < −1 for downregulated and log_2_FC > 1 for upregulated was used to identify candidates of which abundance was changed by the treatments. Candidates that were downregulated in two of three replicates (median log_2_FC < −1) were selected as consistently reduced targets. Proteins that were consistently reduced in all three treatment conditions were targets involved in the rapid ASM-dependent invasion pathway (see Fig. 2D).

### Modeling the APEX2-YFP-CWT construct

The APEX2-YFP-CWT construct was modeled with i-TASSER online algorithm (78–80). The model with the best *C*-score (*c* = −3.30) was used for analysis in Chimera (81).

### Statistical analysis

Statistical analysis was performed in GraphPad prism (V10.1.2). One-sample *t*-test was used for the analysis of normalized data sets. Otherwise, one- or two-way analysis of variance (ANOVA), dependent on the number of variables, was used in combination with suitable multiple comparisons testing. Details about sample size and deployed statistical analysis can be found in respective figure legends. All data are shown as mean ± SD.

## Data Availability

The mass spectrometry proteomics data have been deposited to the ProteomeXchange Consortium via the PRIDE (https://www.ebi.ac.uk/pride) partner repository with the data set identifier PXD051937.

## SUPPLEMENTAL MATERIAL

File S1 mbio.03654-24-s0001.xlsx Identified proteins.

File S2 mbio.03654-24-s0002.xlsx Differentially identified proteins upon host cell treatment.

Supplemental Information mbio.03654-24-s0003.pdf Supplemental figures and captions for Files S1 and S2.

ASM does not own the copyrights to Supplemental Material that may be linked to, or accessed through, an article. The authors have granted ASM a non-exclusive, world-wide license to publish the Supplemental Material files. Please contact the corresponding author directly for reuse.
