# Supplementary material for: Identification of the Staphylococcus aureus endothelial cell surface interactome by proximity labeling
Source: mBio. 2025 Mar 31;16(5):e03654-24. doi: 10.1128/mbio.03654-24 (PMC12077161; doi:10.1128/mbio.03654-24)
Supplement: Supplemental Information — Supplemental figures and captions for Files S1 and S2. [file mbio.03654-24-s0003.pdf]

Supplementary information

A

pathway	description	count in network	strength	signal	false discovery rate
hsa04141	Protein processing in endoplasmic reticulum	20 of 163	0.9	1.63	1.04e-09
hsa05132	Salmonella infection	15 of 209	0.67	0.86	3.28e-05
hsa04510	Focal adhesion	19 of 195	0.8	1.31	5.18e-08
hsa03040	Spliceosome	19 of 132	0.97	1.75	5.68e-10
hsa05205	Proteoglycans in cancer	17 of 194	0.75	1.10	1.26e-06
hsa04810	Regulation of actin cytoskeleton	16 of 209	0.7	0.94	1.01e-05
hsa05012	Parkinson disease	17 of 236	0.67	0.92	1.01e-05
hsa05020	Prion disease	17 of 263	0.62	0.83	2.93e-05
hsa04145	Phagosome	14 of 141	0.81	1.08	3.71e-06
hsa04514	Cell adhesion molecules	14 of 138	0.82	1.09	3.22e-06
hsa04612	Antigen processing and presentation	13 of 64	1.12	1.72	1.15e-08
hsa05130	Pathogenic Escherichia coli infection	12 of 187	0.62	0.65	0.00068
hsa05418	Fluid shear stress and atherosclerosis	12 of 129	0.78	0.92	3.17e-05
hsa04670	Leukocyte transendothelial migration	12 of 111	0.85	1.04	1.01e-05
hsa05412	Arrhythmogenic right ventricular cardiomyopathy	12 of 77	1.0	1.34	5.72e-07

B

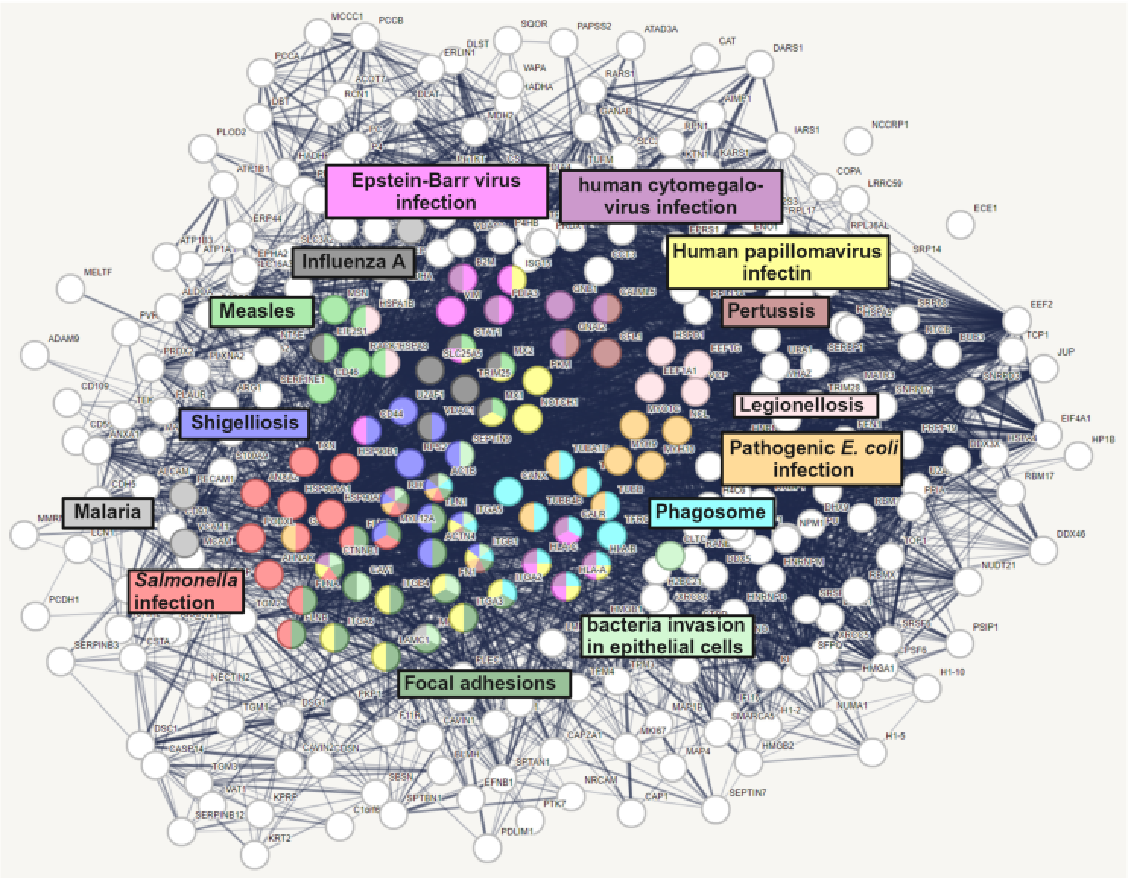

Supp. Figure 1 *S. aureus*-host interactome is a highly connected network containing proteins associated with infection.

Proteins that were identified by APEX2-based proximity labeling as *S. aureus* interaction partners were analyzed via StringDB. Statistical analysis of categories identified in **Figure 1, F** was performed (A). The resulting network (B) has a PPI enrichment p-value < $1x10^{-16}$. Several proteins that were previously associated with infections for instance, bacteria invasion in epithelial cells (FDR $1x10^{-5}$), phagosome (FDR $3.75x10^{-6}$), Shigellosis (FDR 0.0019), Malaria (FDR 0.0308), Salmonella infection (FDR $3.28x10^{-5}$) , pathogenic E.coli infection (FDR 0.00068), Legionellosis (FDR 0.0026), Pertussis (FDR 0.0079), human papillomavirus infection (FDR $9.22x10^{-5}$), human cytomegalovirus infection (FDR 0.0053), Epstein-Barr virus infection (FDR 0.0072), Influenza A (FDR 0.0227), Measles (FDR 0.0031) as well as focal adhesions (FDR $5.18x10^{-8}$), the entry site of *S. aureus* into host cells.

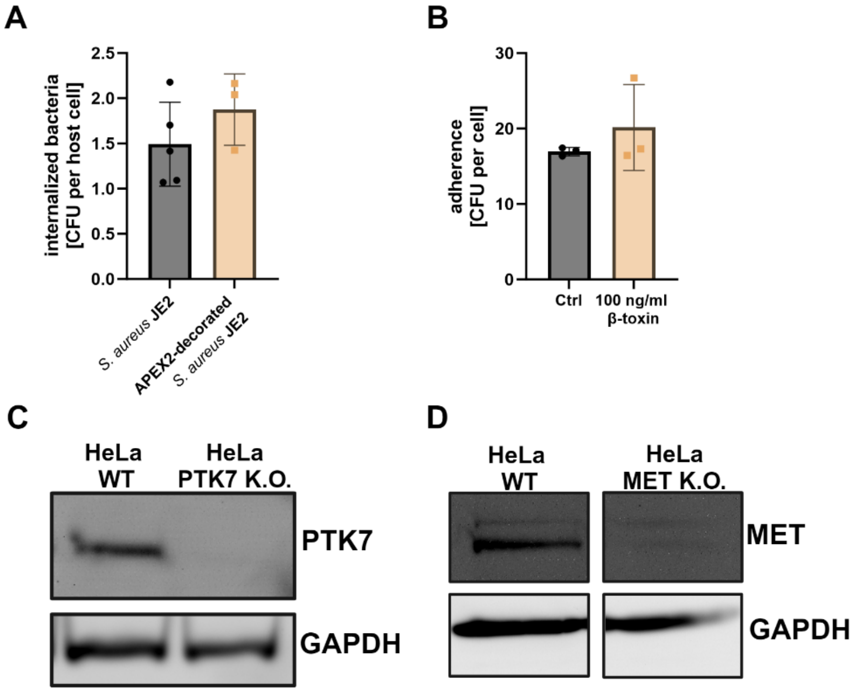

Supp. Figure 2 Pretreatment with bacterial sphingomyelinase does not affect the adherence of *S. aureus* and validation of PTK7 and Met K.O. cell lines.
(A) APEX2-decorated bacteria can invade host cells. HuLEC were either infected with untreated or APEX2-decorated *S. aureus* JE2 at MOI10 for 30 min, extracellular bacteria were removed with lysostaphin, and number of intracellular bacteria was determined via CFU count. CFUs were normalized to number of host cells. **(B) β-toxin treatment does not affect the adherence of APEX2-decorated bacteria to host cells.** HuLEC were treated with β-toxin and infected with APEX2-decorated bacteria at MOI=50. The number of bacteria that adhered to host cells was determined by CFU plating **(C, D) Western blot demonstrates absence of PTK7 or MET in cas9-treated HeLa cell pool.** HeLa WT cells or a HeLa cell pool lacking PTK7 (C) or MET (D) generated by CRISPR-Cas9 were analyzed for the presence of PTK7 or MET by Western blot.

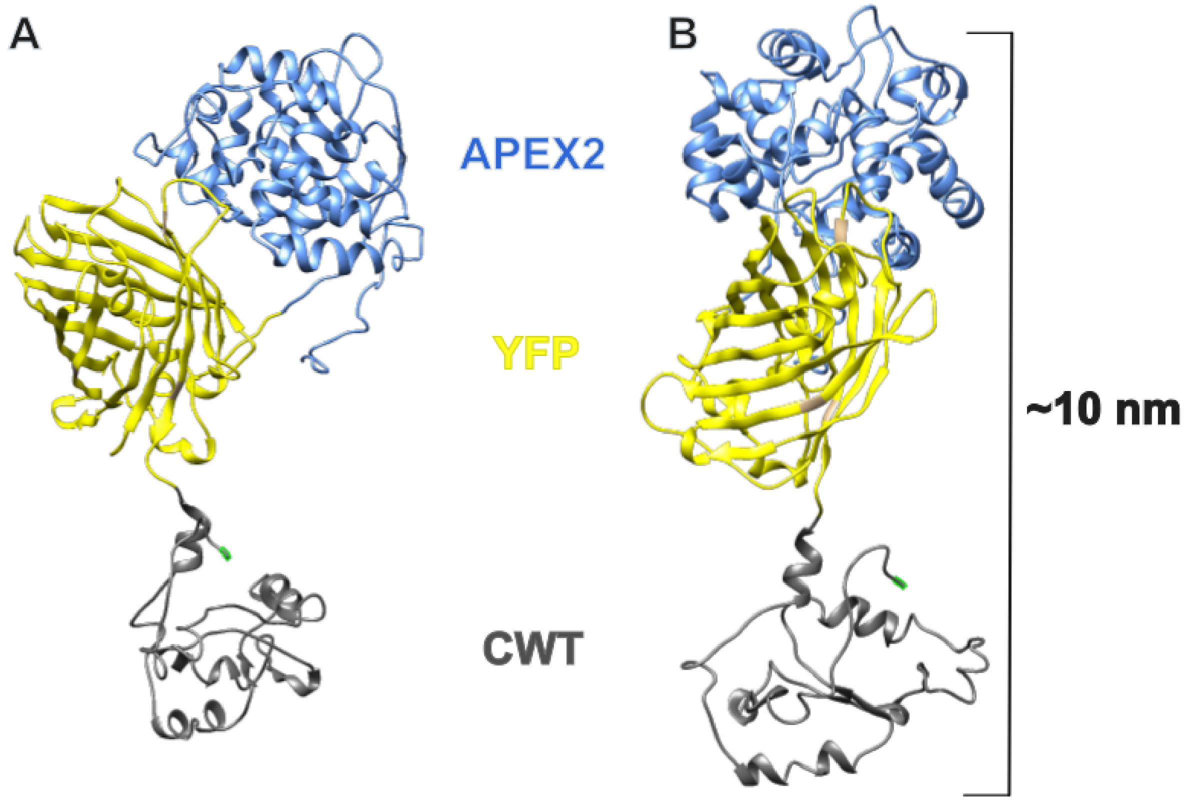

Supp. Figure 3 Model of the protein structure of APEX2-YFP-CWT construct

The protein sequence of the APEX2-YFP-CWT construct was modelled with the i-tasser online algorithm [1-3]. Model was analyzed for lengths (~10 nm) with Chimera software [4]. A and B show the model with 90° rotation along the y-axis. The model has a C-score of -3.30.

Extended Data Legends

Extended Data 1 *S. aureus*-host interactome with annotation and literature research
List of proteins identified in all three replicates of APEX2 proximity labeling. Literature was researched for known interactions of these proteins with pathogens as well as if these proteins are present on the host cell surface.

Extended Data 2 List of proteins with altered *S. aureus* interaction upon ionomycin, β-toxin and amitriptyline treatment of host cells
List of proteins whose abundance was affected by amitriptyline, β-toxin or ionomycin treatment. Log2 foldchanges are indicated for individual replicates. Proteins that were affected by all three conditions were selected.

Supplementary References

1. Yang, J., et al., *The I-TASSER Suite: protein structure and function prediction.* Nature Methods, 2015. **12**(1): p. 7-8.
2. Roy, A., A. Kucukural, and Y. Zhang, *I-TASSER: a unified platform for automated protein structure and function prediction.* Nature Protocols, 2010. **5**(4): p. 725-738.
3. Zhang, Y., *I-TASSER server for protein 3D structure prediction.* BMC Bioinformatics, 2008. **9**(1): p. 40.
4. Pettersen, E.F., et al., *UCSF Chimera--a visualization system for exploratory research and analysis.* J Comput Chem, 2004. **25**(13): p. 1605-12.
